# Effect of exosomal miRNA on cancer biology and clinical applications

**DOI:** 10.1186/s12943-018-0897-7

**Published:** 2018-10-11

**Authors:** Zhenqiang Sun, Ke Shi, Shuaixi Yang, Jinbo Liu, Quanbo Zhou, Guixian Wang, Junmin Song, Zhen Li, Zhiyong Zhang, Weitang Yuan

**Affiliations:** 1 Department of Anorectal Surgery, The First Affiliated Hospital of Zhengzhou University, Zhengzhou, 450052 Henan China; 2 Department of Plastic Surgery, The First Affiliated Hospital of Zhengzhou University, Zhengzhou, 450052 Henan China

**Keywords:** Exosomal miRNAs, Cancer, Metastasis, Angiogenesis, Biomarkers

## Abstract

Exosomes, extracellular vesicles with diameters ranging from 30 to 150 nm, are widely present in various body fluids. Recently, microRNAs (miRNAs) have been identified in exosomes, the biogenesis, release, and uptake of which may involve the endosomal sorting complex required for transport (ESCRT complex) and relevant proteins. After release, exosomes are taken up by neighboring or distant cells, and the miRNAs contained within modulate such processes as interfering with tumor immunity and the microenvironment, possibly facilitating tumor growth, invasion, metastasis, angiogenesis and drug resistance. Therefore, exosomal miRNAs have a significant function in regulating cancer progression. Here, we briefly review recent findings regarding tumor-derived exosomes, including RNA sorting and delivering mechanism. We then describe the intercommunication occurring between different cells via exosomal miRNAs in tumor microenvironmnt, with impacts on tumor proliferation, vascularization, metastasis and other biological characteristics. Finally, we highlight the potential role of these molecules as biomarkers in cancer diagnosis and prognosis and tumor resistance to therapeutics.

## Background

In recent years, researchers and clinicians have mostly focused on the identification of cancer-specific targets and the development of targeted therapies that may efficiently kill cancer cells. Although considerable success has been achieved with regard to identifying effective small cancer-specific targets and a series of monoclonal antibodies [1]. However, obvious drawbacks exist. For example, cancers are characterized by extensive heterogeneity and a variety of subtypes, which complicates the identification of unique targets and the eradication of all tumor cells, due to clonal evolution of malignant cells. Another unresolved problem is how to increase the efficiency and accuracy of cancer-specific target molecules when delivered. In depth research of extracellular vesicles, especially exosome (30–100 nm), raised the intriguing possibility that exosomal cargo may be a good way to protect target molecules integrity and to enhance the accuracy of delivery [2, 3]. Cancer cells secrete at least 10-fold more exosomes than do normal cells, and tumor-derived exosomes (TDEs) can facilitate cell-cell communication through the transport of growth factors, chemokines, microRNAs, and other small molecules [4, 5]. Moreover, profiling studies have revealed that exosomes of different cellular origin contain a unique expression profile of mRNAs and miRNAs, which may also differ from the signatures of their parent cells [6]. What’s more, accumulating evidence suggests that tumor microenvironment highly contributes to metabolic rewiring of cancer cells via extracellular microvesicles, this fosters complete nutrient exploitation and favors OXPHOS of lipids and glutamine at the expense of glycolysis, thereby changing the microenvironment from a normal state to a tumor-favorable state that allows for tumor growth, invasion, and drug resistance [7]. miRNA-carrying exosomes released from immune cells, mesenchymal cells and cancer cells in the tumor environment can shuttle from donor cells to recipient cells [8, 9]. In addition, cancer-derived miRNA-exosomes contribute to the recruitment and reprogramming of constituents associated with tumor environment [10]. Therefore, exosomal miRNAs are likely to be applied as promising non-invasive biomarkers and potential targetable factors in cancer diagnosis and treatment.

### The biogenesis, release, and uptake of exosomes and exosomal miRNAs

Exosomes are nano-vesicles present in the circulation that are involved in cell-to-cell communication and regulation of different biological processes. miRNAs are part of their cargo and are potential biomarkers [11]. As exosomes carry proteins, mRNAs and miRNAs that can be transferred from donor to recipient cells via target cell membrane fusion, these vesicles have recently been recognized as important mediators of interactions between different cells [2]. In tumor microenvironment, the process described above is indispensable for the transfer of cancer-promoting cellular contents to surrounding cells, thereby accelerating cancer progression [12]. During this process, the transfer of exosomal microRNAs to recipient cells to regulate target gene expression is particularly attractive, and knowledge of the biogenesis, release, and uptake of exosomes and exosomal miRNAs is helpful for both understanding the biological mechanism of cancer progression and further exploring therapeutic approaches [13].

Accumulating evidence supports that the biogenesis, uptake and material cargo sorting of exosomes involve the endosomal sorting complex required for transport (ESCRT complex) and relevant proteins [14]. The ESCRT complex can select the “cargo” protein labeled by ubiquitin, direct it to multivesicular bodies(MVBs), and then separate fromthe peripheral membrane in a highly conserved process that is homologous to the process of cytokinesis and virus budding [15]. Study of late endosome components, such as Alix, tumor susceptibility gene 101 (TSG101) and tetraspanins, promotesanunderstanding of exosomal origin [16].

**Fig. 1:**
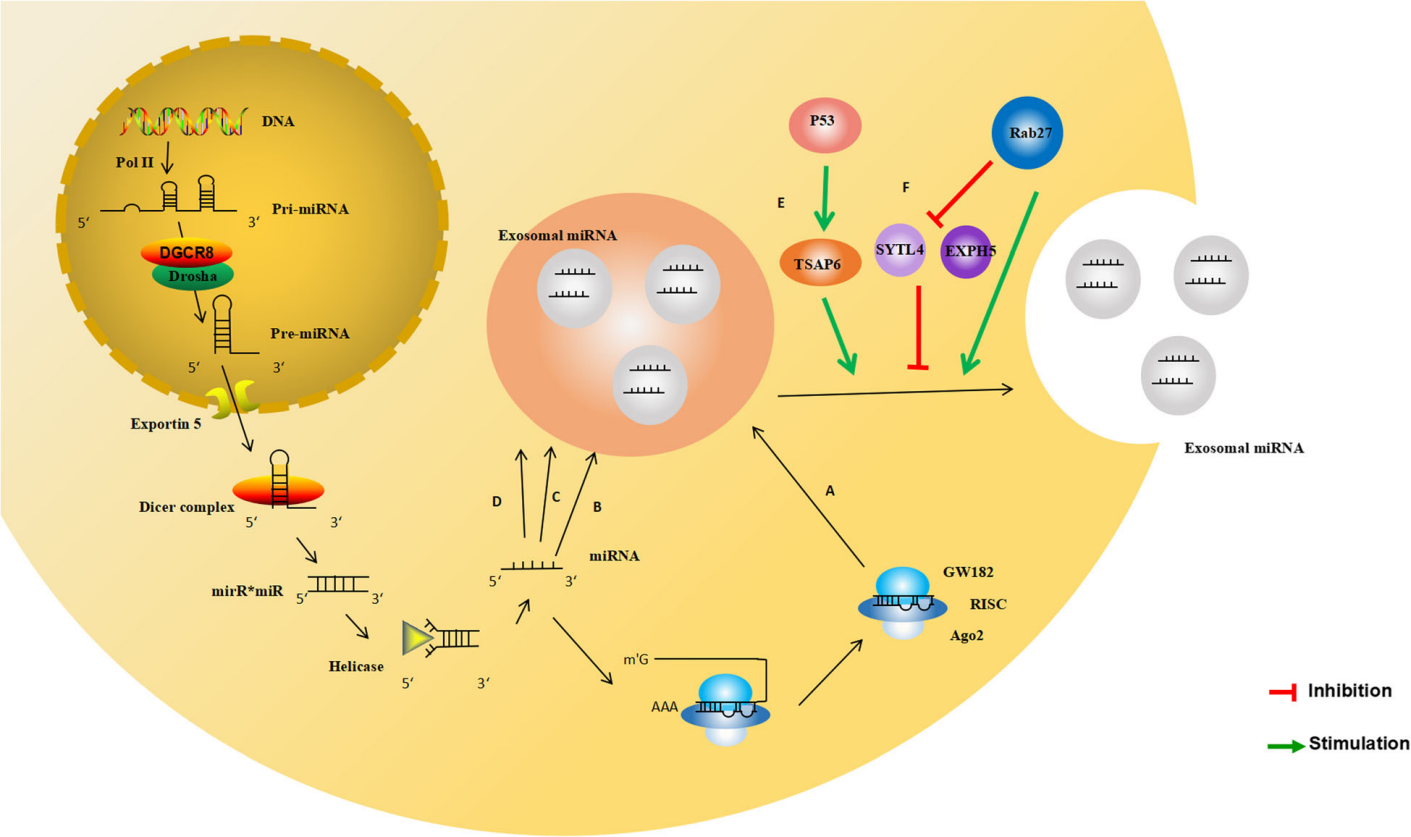
The sorting mechanism of exosomal miRNA MiRNA genes are transcribed into primary miRNAs (pri-miRNA) by Pol-II. Then with the catalytic action of DGCR8 and Drosha complex, pri-RNA are transmitted into pre-miRNA, which are exported out of the nucleus by exportin5 complex. In the cytoplasm, the pre-miRNAs are digested by the Dicer complex into double-stranded miRNAs, which turn to be single-stranded ones, mature miRNAs, in the next step by Helicase. Mature miRNAs are sorted into exosomes via four potential modes: **a** the miRISC-related pathway; **b** nSMase2-dependent pathway; **c** miRNA motif and sumoylated hnRNPs-dependent pathway; **d** 3’miRNA sequence-dependent pathway. **e** Knockdown of Rab27 or their effectors, SYTL4 and EXPH5, could inhibit secretion of exosomes in HeLa cells. **f** Both the tumor repressor protein p53 and its downstream effector TSAP6 could enhance exosome production

Interestingly, it has recently been reported that miRNAs in a precursor state (pre-miRNA) associated with the processing complex (e.g., Dicer, Ago2 and TRBP) can be found inside breast cancer-derived exosomes, where they are processed into mature miRNAs, establishing a new method by which miRNAs are integrated into exosomes. In this scenario, the formation and activation of exosomal miRNAs needs to be stressed [17]. Canonically, the biogenesis of miRNAs begins in the nucleus where DNA containing miRNAs is transcribed by RNA polymerase II to generate primary miRNAs (pri-miRNAs) (Fig. 1).These pri-miRNAs are first transcribed as parts of longer molecules, up to several kilobases in length, which are processed in the nucleus into hairpin RNAs of 70–100 nt by the double-stranded RNA-specific ribonuclease, Drosha [18]. Hairpin pre-miRNAs are then transported by exportin 5 to the cytoplasm, where they undergo further processing by a double-stranded-specific ribonuclease, called Dicer. After maturation, double-stranded miRNAs converted into single-stranded miRNAs, and mature miRNAs are sorted into exosomes via different modes. In the miRISC-related pathway, a representative mode, single-stranded miRNAs are incorporated into RNA-induced silencing complex (RISC) along with argonaute (AGO2) and GW182, and primarily bind to specific messenger RNAs (mRNAs) at specific sequence motifs, predominantly within the 3′ untranslated region (3′UTR); these motifs are significantly, though not completely, complementary to the miRNA. The mRNA/miRNA duplex then inhibits translation by blocking initiation or enhancing degradation of the mRNA [19]. Finally, the MVBs fuse with the cell membrane and release the intraluminal endosomal vesicles into the extracellular space, which then become exosomes. There are some studies indicate that some molecules act as a regulatory network and is responsible for the formation and secretion of exosomes in parent cells. For instance, Rab27a and Rab27b were found to function in multivesicular endosomes (MVEs) docking at the plasma membrane. The size of MVEs was strongly influenced by Rab27a and Rab27b silencing. With knockdown of Rab27 or its effectors SYTL4 and EXPH5 inhibiting secretion of exosomes in HeLa cells [20, 21]. In addition, a set of proteins encoded by genes that are not transcriptional targets of p53 were found to exit the cell via exosomes and exosome production by cells was found to be regulated by the p53 response. Its downstream effector TSAP6 was shown to enhance exosome production in cells undergoing a p53 response to stress. Thus, the p53 pathway regulates the production of exosomes into the medium [22]. Moverover, syndecan-syntenin interact directly with the ALIX protein via Leu-Tyr-Pro-X(n)-Leu motif to support the intraluminal budding of endosomal membranes, which is an important step in exosome formation [23, 24].

Rab27a and Rab27b have been reported to be associated with exosome secretion, with knockdown of Rab27 or its effectors SYTL4 and EXPH5 inhibiting secretion of exosomes in HeLa cells. In addition, both the tumor repressor protein p53 and its downstream effector TSAP6 enhance exosome production. Moreover, syndecan-syntenin interact directly with the ALIX protein via Leu-Tyr-Pro-X(n)-Leu motif to support the intraluminal budding of endosomal membranes, which is an important step in exosome formation. All of these studies indicate that a set of molecules act as a regulatory network and is responsible for the formation and secretion of exosomes in parent cells.

### Experimental methodology of isolating exosomal miRNAs

For implementation of the use of new biomarkers into clinical practice, the first step is to standardize exosomal measurement and to evaluate their stability. However, there is not a gold standard for exosome isolation. Thus far, ultracentrifugation was the popular methodology applied for their isolation because it was reproducible and could be provided optimal amounts of exosomes. The different centrifugal force and duration to isolate exosomes are easy to control, based on their density and size differences from other components in a sample, consisting of serum isolation with 100,000 g to 120,000 g, urine exosome isolation with 17,000 g, and milk exosome isolation with 12,000 g to 35,000 g. While the disadvantages prevent its effectiveness, including excessive pressure suffered by exosomes during this process, lack of specificity during the precipitation, excessive time, the equipment required for isolation, and difficulties in exactly reproducing the isolation in different places. Another isolation method commonly used is size exclusion chromatography. It allows a better degree of purity and is less harmful to exosomes. Nevertheless, the high final dilution of the exosome sample makes it difficult to use them in downstream applications that require a high exosome concentration, such as the evaluation of their miRNA profile. Finally, during recent years, there has been an increase in the number of commercial kits developed for exosome isolation. Most of them are based on precipitation. Although they are not completely specific and precipitate some impurities, their rapidity and reproducibility even in different labs make them useful for future diagnosis, primarily in miRNA-based tests. Other recent publishment pointed out the importance of freezing plasma before exosome isolation, RNA isolation and qPCR for miRNAs rather than freezing exosomes before miRNA analysis, by comparing the miRNA levels obtained from exosomes isolated from fresh plasma with that from frozen one. And it was necessary to determine the inter- and intra-individual variability of healthy subjects, which could help to optimize sample size in future studies with circulating exosomes. After isolating exosomes, some researchers have developed methods for exploiting differences between tumor-associated and non-tumor exosomes surface composition. For instance, detecting cancerous exosomes from SKOV-3 ovarian tumor cells in real time by the technique of multi-parametric surface plasmon resonance (MP-SPR) to measure LXY30 binding, without a priori labeling.

As for the experimental skill of exosomal miRNAs examination and protection, he current commonly avenue is quantitative reverse transcription polymerase chain reaction (qRT-PCR), however, this method requires highly trained experience and have the potential to generate false positive signals. Later, some groups developed PCR-free methods for exosomal miRNAs quantitation based on ratiometric electrochemistry, localized surface plasmon resonance (LSPR), and surface-enhanced Raman scattering (SERS), respectively, while the expensive instrument and complex operation have hampered their extensive application. At present, fluorescent methods have been given attention, because of their intrinsic advantages, including simple instrumentation, as well as high sensitivity and capacity to high-throughput screening.To date, several attempts have been reported using fluorescent methods to detect exosomal miRNAs with various degrees of success, as evidenced by the cationic lipoplex nanoparticles containing a molecular beacon assay, fluorescent dye-labeled molecular beacons strategies, fluorescence signal amplifiable biochip assay, and others. However, these methods employed solely responsive signal and were based on measuring the absolute change of the fluorescent intensity, which was readily perturbed by numerous experimental conditions, including thermodynamic fluctuations, nuclease degradation, and dye photobleaching. To utilize exosomal miRNAs as a diagnosis biomarker, a fluorescent system with antidisturbance should be developed, due to the complex biosystem. Surprisingly, because of the self-referencing capability, ratiometric fluorescent measurement is able to cancel out environmental fluctuations by calculating the emission intensity ratio at two different wavelengths. Recently, the practical applications of ratiometric fluorescent bioprobes has been improved. For instance, a ratiometric fluorescent bioprobe based on DNA-labeled carbon dots (DNA-CDs) and 5,7-dinitro-2-sulfo-acridone (DSA) coupling with the target-catalyzing signal amplification for the detection of exosomal miRNA-21. There was high fluorescence resonance energy transfer (FRET) efficiency between carbon dots (CDs) and DSA when the bioprobe was assembled.

After gain the exosomal miRNAs, some researches claim that a new concept for miRNA editing measurement would be necessary, which considered not only the absolute editing level of miRNA but the miRNAs modification assessed via reads per million reads mapped to miRNAs (RPM). For example, by analyzing small-RNA sequencing data from exosome samples of NSCLC patients at different stages, researchers found that editing(ED) miR-411–5p downregulated, while wild-type (WT) showed no significant difference in expression. Further study showed that miR-411–5p edited in position 5 was differentially expressed between NSCLC and normal tissue samples, indicating that the machinery that governs the export of miRNAs to extracellular space in tumor conditions may discriminate ED miRNAs differently. Thus, they thought post-transcriptional modifications in miRNAs within both tissues and circulation could both serve as potential novel biomarkers and provide additional insights into the pathogenesis of cancers.

### Exosomal miRNA in Cancer

**Fig. 2:**
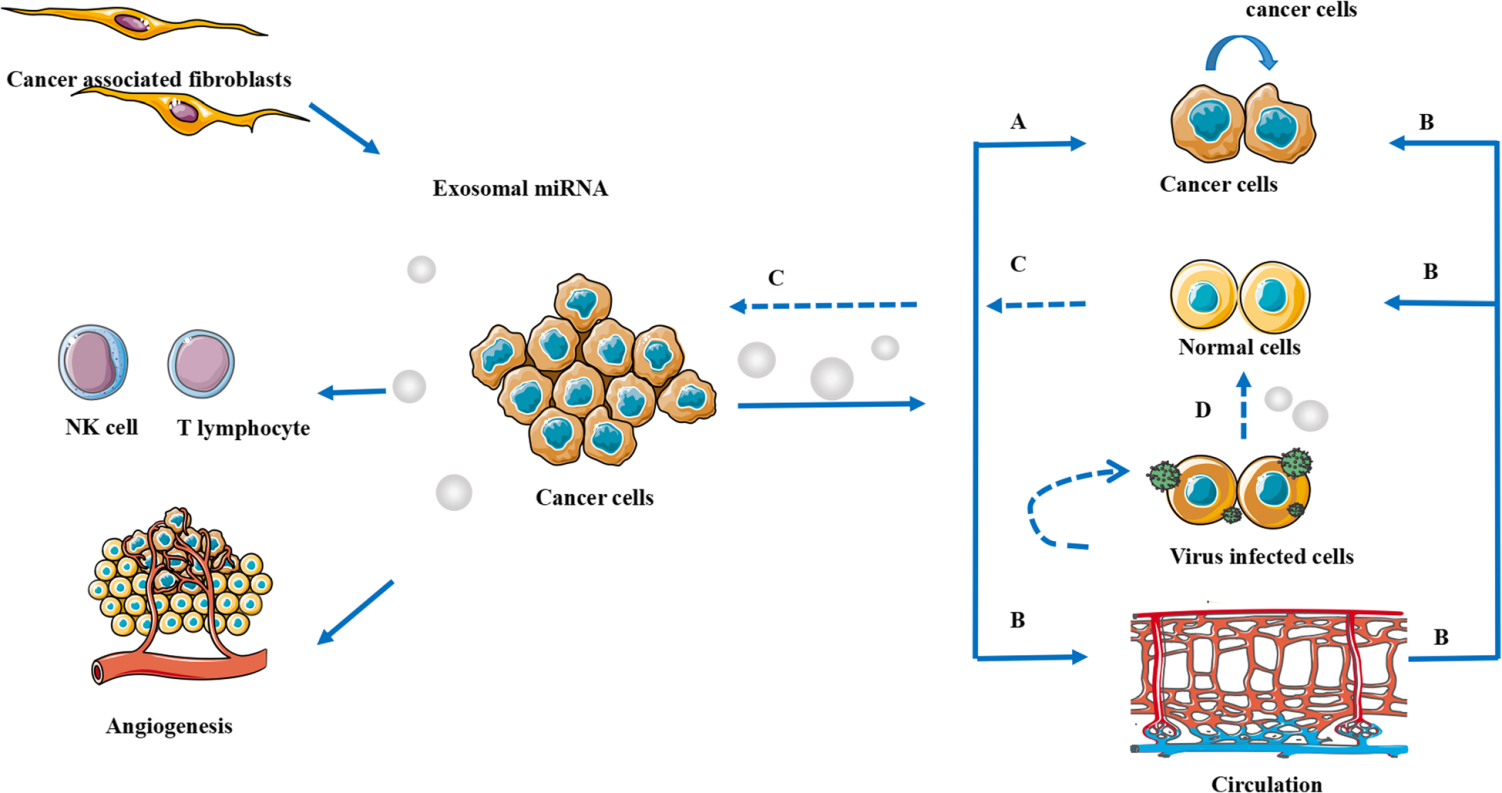
Exosomal miRNA in Cancer. **a** The first general mechanism is that cancer cells export exosomal miRNA to parent surrounding cancer cells. **b** The second general mechanism is that primary tumor cells can communicate with other cells via exosomal miRNAs in the tumor microenvironment. **c** The third general mechanism is that exosomes derived from normal cells alter the behavior of tumor cells. **d** The forth general mechanism is that exosomes derived from cells infected with virus to influencr normal cells oncology and themselves

The malignant phenotypes of tumors are not only determined by cancer cells themselves but also depend on the surrounding tumor microenvironments [25]. Studies on the relationship between exosomal miRNAs and cancer begin to reveal a general picture of their ubiquitous involvement in cellular pathways from life to death, from metabolism to communication. These molecules have an undeniable role in cancer both as tumor suppressors and promoters modulating cell proliferation and migration, the epithelial-mesenchymal transition (EMT), and tumor proliferation, angiogenesis and metastasis [5]. Moreover, exosomal microRNAs can even affect the environment surrounding the tumor, influencing the extracellular matrix (ECM) as well as immune system activation and recruitment. Clearly, the influence of exosomal miRNA on cancer is somewhat similar to that of miRNA [23] (Fig. 2).

### miRNAs, ECM, and Cancer-associated fibroblasts (CAFs) miRNAs and ECM

The tumor microenvironment is defined as the variety of normal cells, blood vessels, signaling molecules, and ECM that surround tumor cells [19]. The cellular components of the tumor microenvironment include endothelial cells, pericytes, fibroblasts, and immune cells [26]. Both tumor environmental cues and cell-intrinsic alterations contribute to these epigenetic changes, inducing adaptations by cancer cells that allow successful invasion of the stroma, entry and survival in lymphatic or blood vessels, spread to and colonization of distant/different organs, as well as resistance to cytotoxic drugs [27]. Cancer-associated fibroblasts(CAFs) are vital constituents of the tumor microenvironment, and their interactions with cancer cells play a major role in mediating their formation and activation [28, 29].

CAFs isolated from cancer patients have a morphology and function that differs from that of normal fibroblasts (NFs). CAFs have been shown to promote the invasion and growth of tumor cells [30]. CAFs produce growth factors (e.g., vascular endothelial growth factor (VEGF)) and cytokines (e.g., TGFβ, IL-6, IL-10) that activate the adjacent ECM, contributing to cancer cell growth. Additionally, CAFs are the primary source of an altered ECM, containing fibronectin and collagen, and also promote tumor growth [31]. CAF-secreted factors include proinflammatory cytokines, typically IL-1β and IL-8 typically, which are associated with pro-tumorigenic effects. SDF-1α, a prominent chemokinesecreted by CAFs, promotes proliferation, by signaling through chemokine (CXC) receptor 4 (CXCR4) [32]. NFs have been shown to inhibit tumor growth, unlike CAFs, and it has recently been reported that exosomal miRNAs might convert NFs into CAFs for tumor survival. Nonetheless, how this communication promotes activation of NFs into CAFs remains poorly understood.

Recent studies have demonstrated that pancreatic cancer cells secrete exosomal miR-155 to activate NFs. This phenomenon might be related to miR-155-mediated downregulation of its target TP53INP1 [33]. Moreover, previous studies have shown that highly metastatic hepatocellular carcinoma (HCC) cells secrete exosomal miR-1247-3p targeting B4GALT3, leading to activation of β1-integrin-NF-kB signaling in fibroblasts. Activated CAFs further promote cancer progression by secreting proinflammatory cytokines, including IL-6 and IL-8 [34]. In addition, the relationship between exosomal miRNAs and CAFs activation is unlikely to be unidirectional. A CAF-like phenotype inducible by tumor cells through exosome-mediated delivery of miR-9 was reported in triple-negative breast cancer. Interestingly, miR-9 is also released by NFs and transferred to tumor cells [30]. All of these studies indicate that exosomal miRNA and their targets act as a regulatory network responsible for transformation of the tumor microenvironment.

### Exosomal miRNAs and tumor immunity

Emerging evidences suggests that tumor-derived exosomes participate in tumor immune escape by delivering immunosuppressive molecules and factors [35]. Exosomal miRNAs are carriers of information that is able to reprogram functions of immunologically active factor and immune target cells, such as dendritic cells (DCs), natural killer (NK) cells, and T lymphocytes et al. [36].

It has been shown that proinflammatory conditions might promote tumorigenesis [37]. DCs are crucial regulators of the immune system that initiate immunity or immunological tolerance depending on their state of activation [38].When activatedupon exposure to danger signals from pathogens or damaged tissue, DCs trigger the activity of pattern recognition receptors, such as Toll-like receptors (TLRs) [39]. Upon TLR stimulation, DCs upregulate costimulatory molecules and proinflammatory cytokines to stimulate T lymphocytes and initiate immune responses [40]. Non-small cell lung cancer (NSCLC) secretes an abundance of exosomes containing miR-21 and miR-29a, which can bind to TLRs to induce protumoral inflammation, leading to tumor growth and metastasis [41]. Overexpression of miR-203 in pancreatic adenocarcinoma has a similar effect on TLR4 as miR-21 and miR-29a [42]. Moreover, pancreatic cancer-derived exosomes transfer miRNAs toDCs and inhibit Regulatory factor X-associated protein(RFXAP) expression via miR-212-3p, inducing MHC II downregulation and immune tolerance of DCs [36].

Exosomal miRNAs also play a role in the biology of NK cells and T lymphocytes. NKs are a sub-population of T cells with a role as tumor cell killer, which can produce a series of antitumor cytokines, including IL-4, IFN-γ, FasL, IL-13, and perforin [43]. Importantly, their efficiency is abrogated by exposure to TGF-β. Meanwhile, TGF-β-inducible miR-183 silences tumor-associated natural killer cells by targeting and repressing DNAX activating protein [44]. Moreover, hypoxia-inducible miR-210 regulates the susceptibility of tumor cells to lysis by cytotoxic T cells. Hypoxic tumor-derived microvesicles negatively regulate NK cell function by a mechanism, involving TGF-β and miR-23a transfer [45].

Besides, the process, exosomic miRNAs acting on NKs immune activity and then inducing tumor resistance to immunology, involves in many-sided, many-targeted, many-factored effect. Here we focus on some emblematical miRNAs from TDE shown in Table 1.

**Table 1:** miRNAs involved in the line of communication cancer-immune

Immuno	Exosomal miRNA	Involved molecule	Involved other molecules	Function	Ref.
DCs	miR-203	TLR4	TNF-α, IL-12 pathway	DCs dysfunction in pancreatic cancer	[42]
	miR-212-3p	RFXAP	/	Immune tolerance of DCs in pancreatic cancer	[36]
Lymphocytes	miR-183	DAP12	TGFβ	NK	[94]
	miR-210	NKG2D	TGFβ1	NK	[45]
	miR-23	CD107a	/	NK	[95]
	miR-20a	MICA/MICB	NKG2D	NK	[96]
	miR-10b	MICB	/	NK	[97]
	miR-92a	/	FasL, INF-ϒ	NKT	[98]
	miR-214	PTEN	IL-10	T cell	[99]

### Exosomal miRNA and tumor proliferation

Malignant cells have the ability to transfer genetic information to other cells in the tumor microenvironment through exosomes. Some of the exosomic miRNAs transported between donors and recipients are shown in Table 2, indicating that exosomal miRNAs contribute to cancer cell proliferation, angiogenesis, metastasis, drug resistance and tumor inhibition.

**Table 2:** Exosomal miRNAs as prognostic and predictive biomarkers

Systematic		Cancer type	Exosomal miRNAs	Donor	Recipient	Target(s)	Function	Type of biomaker	Ref.
Respiratory system		Lung cancer	miR-155/ -146a	Immune cells	Immune cells	HO1/ IRAK1 and TRAF6 TP532NP1	MiR-155 enhances while miR-146a reduces inflammatory gene expression. Promotes endotoxininduced inflammation.	Inflammation	[10]
			/	Mast cell	KIT-SCF/ PI3K	/	Enhances proliferation in recipient tumor cells.	Proliferation	[100]
			miR-210	Lung adenocarcinoma	Stromal cells	Ephrin A3	Promotes angiogenesis.	Angiogenesis	[52]
			miR-21	Bronchial epithelial (HBE) cells	Normal HBE cells	STAT3	Increases VEGF levels in recipient cells, which is involved in angiogenesis and malignant transformation of HBE cells.	Angiogenesis	[53]
			miR-192	A549	Endothelial cells	ICAM-1/ PTPRJ	Regulates non-cell-autonomous invasiveness, and tumor-induced osteoclastogenesis.	Bone metastasis	[100]
			miR-494	Lung adenocarcinoma cells	Lymph nodes, lung cells	MAL,cdh17	/	Pre-metastasis	[101]
			miR-542-3p			cdn17, TRAF4			
			miR-23b-3p, miR-10b-5p and miR-21-5p	Plasmatic exosomes	Non-small cell lung cancer cells(NSCLC)	/	/	Progression, angiogenesis and metastasis	[102, 103]
Digestive system	Digestive tract	Esophageal cancer(ESCC)	miR-30a	ESCC cells	/	WNT2/FZD2	Down-regulation of miR-30a-3p/5p expression is correlated with the activation of Wnt signaling in ESCC, which enhances cell proliferation.	Proliferation	[104]
		Gastric cancer	miR-21	Macrophage	BGC-823	PDCD4	MiR-21 inhibitor-loaded exosomes promote migration and reduce apoptosis.	Metastasis	[57]
			miR-221	Mesenchymal stem cells	HGC-27	/	Promotes HGC-27 growth and migration.	Metastasis	[105]
		Colorectal cancer(CRC)	miR-21, −192 and − 221	HCT-15, SW480 and WiDr	HepG2 and A549	/	Regulate the expression of target genes in HepG2 and A549 cells. May promote various functions.	/	[73]
			Let-7a	CRC cells	T cells	/	Let-7a expression is positively associated with cancer-specific mortality, and T cells low expresion.	Inmmue inhibitor	[106]
			miR-19a	CRC cells	/	PTEN	Over-expression was significantly associated with poorer survival.	Metastasis	[76]
			miR-23b-3p	Blood plasma isolated from CRC patients	Colon cancer cells	/	/	Inhibitor	[103]
	Digestive gland	Liver cancer	miR-142 and − 223	Macrophages	Hepatocellular carcinoma cells (HuH7 and HepG2)	Stathmin-1/ IGF1R	Inhibits proliferation of cancer cells.	Inhibitor	[77]
			miR-122	Huh7 cells	HepG2 cells	IGF1R mRNA	Reduced growth and proliferation of recipient HepG2 cells.	Inhibitor	[107]
			miR-584	Hep3B, HepG2, and PLC/PRF/5	Hep3B, HepG2 and PLC/PRF/5	TGF-β activated kinase-1 (TAK1)	HCC cell-derived exosomes modulate TAK1 expression and associated signaling. They also enhance the growth of transformed recipient cells.	Proliferation	[47]
		Pancreatic cancer (PC)	miR-122-5p and miR-193b-3p	Plasma samples	Pancreatic cancer cells	/	Act on several molecular pathways closely related with PC such as p53 signaling pathway, TGF-beta signaling pathway and so on.	Proliferation	[108]
			miR-221-3p					Inhibitor	
			miR-23b-3p	PANC-1 cells	PANC-1 cells	CA-19-9	miR-23b-3p expression in sera or that in the exosomes isolated from sera showed a close relationship with CA-19-9 expression.	Proliferation and metastasis	[103, 109]
			miR-141, miR-375	PCa cells	Serum	/	miR-141 and miR-375 were associated with recurrent (metastatic) PCa following radical prostatectomy	metastasis	[110]
			miR-1290, miR-375	PCa cells	Plasma	/	Various RNA species and changes in exosomal RNA contents are robust candidates as clinical biomarkers for advanced PCa	Survival prognosis	[111]
			miR-19b	PCa cells	Urine	/	Active secretion of miR-19b containing vesicles by tumor cells	Diagnosis	[112]
			Isomirs of miR-21, miR-375 and miR-204	PCa cells	Urine	MARCKS, BTG2, PTEN, RECK	The miRNA-read-length of miR-204, miR-21 and miR-375 showed clear differences when comparing controls with PCa patient samples.	Progression	[113, 114]
			miR-141	PCa cells	Serum	/	Exosomal miR-141 is upregulated in the serum from patients with PCa compared with patients with benign prostate hyperplasia or the healthy volunteers	Metastasis	[115]
			miR-200c-3p	Urine	PCa cells	ZEB1, ZEB2, SNAIL2	miRNA re-expression inhibits prostasphere formation, decreases clonogenic survival, and reduces NOTCH1 and LIN28B gene expression, the drivers of self-renewal.	Suppressor	[114, 116]
			miR-21-5p	Urine	PCa cells	MARCKS, BTG2, PTEN, RECK	Blocking miRNA with antisense oligonucleotides has no effect on cell proliferation, but it leads to increased sensitivity to apoptosis and the inhibition of cell motility and invasion	Progression	
			Let-7c	Urine	PCa cells	E2F2 and CCND2, LIN28, MYC, EZH2	miRNA family are down-regulated in PCa.	Suppressor	
			miR-196a-5p	Urine	PCa cells	ETS-related gene (ERG)	The high levels of miR-196a-5p in normal prostate cells help to maintain the levels of ERG low.	Metastasis	[117]
			miR-501-3p	Urine	PCa cells	E-cadherin	miR-501-3p promoted the invasiveness of pancreatic ductal adenocarcinoma cells possibly by suppressing E-cadherin		
			miR-2909	Urine	PCa cells	/	miR-2909 levels were only increased in urinary exosome from PCa patients	Metastasis	[118]
			miR-145	Urine	PCa cells	KRAS, ERK5, KLKs, FSCN1, SWAP70, MMP-13, GOLM1, FNDC3B, CD133, CD44, OCT4, MYC, KLF4	Urinary levels of exosomal miR-145 were increased in PCa patients vs BPH patients	Suppressor	[114, 119]
			miR-1246	Serum	PCa cells	/	Serum levels of exosomal miR-1246 were increased in PCa patients vs BPH patients	Metastasis	[120]
		Cholangiocarcinoma	/	KMBC and HuCCT1	Mesenchymal stem cells	/	Enhance MSC migratory capability and expression of alpha-smooth muscle actin mRNA. Promote the release of CXCL-1, CCL2, and IL-6.	Metastasis	[121]
Urinary system		Bladder cancer	Exosome-derived miR- 29c	miR-29c	BIU-87 cells	BCL-2 and MCL-1	Exosome-derived microRNA29c induces apoptosis in bladder cancer cells by down-regulating BCL-2 and MCL-1.	Apoptosis	[78]
Reproductive system	Female	Breast cancer	miR-105	MDA-MB-231	Endothelial cells	Protein ZO-1	Destroys tight junctions and the integrity of natural barriers to metastasis.	Metastasis	[58]
			miR-10b	MDA-MB-231	HMLE (MCF-7)	HOXD10/KLF4	Induces invasion of non-malignant HMLE cells.	Metastasis	[122]
			miR-210	MDA-MB-231 4 T1	Endothelial cells	/	Suppresses expression of specific target genes resulting in enhanced angiogenesis.	Metastasis	[123]
			miR-503	Endothelial cells	Breast cancer cells	CCND2/ CCND	Alters proliferation and invasion.	Metastasis	[124]
			miR-16	EGCG-treated 4 T1 cells	Macrophages	/	Inhibits TAM infiltration and M2 polarization.	Metastasis	[125]
			miR-16	Mesenchymal stem cells	4 T1	VEGF mRNA	Down-regulates the expression of vascular endothelial growth factor (VEGF) in tumor cells.	Metastasis	[126]
			miR-140	Pre-adipocyte (3T3L1)	MCF10	SOX9	Regulates differentiation, stemness, and migration.	Metastasis	[127]
			miR-122	Breast cancer patients/ MCF10A	Recipient pre-metastatic niche cells	PKM2 and GLUT1	Suppresses glucose uptake by niche cells by down-regulating pyruvate kinase	Metastasis	[128]
				Hepatoma cells (Huh-7 and Hep3B cells)	MCF-7 cells	SDC1	The liver-derived exosomes increased the mobility of breast cancer MCF-7 cells though SDC1 downregulation mediated by exosomal miR-122-5p.	Metastasis	[60]
			miR-23b	Bone marrow mesenchymal stem cells	Breast cancer cells	MARCKS	Decreases MARCKS expression and promotes breast cancer cell dormancy in the metastatic niche.	Dormancy	[129]
			miR-127, −197, −222, and − 223	Bone marrow stroma	MDA-MB-231	CXCL12	Reduce CXCL12 levels and decreases proliferation. Elicit dormancy in bone marrow metastases in breast cancer.	Dormancy	[79]
			miR-134	Hs578T and Hs578Ts(i)8	Breast cancer cells	STAT5B	Reduces STAT5B and Hsp90 expression. Decreases cell migration and invasion.	Drug resistance	[130]
			miR-221/ -222	MCF-7 (Tamoxifen resistant)	MCF-7 (Tamoxifen-sensitive)	P27 and ERα	Enhances tamoxifen resistance in recipient cells.	Drug resistance	[68]
			miR-223	IL-4-activated macrophages	MDA-MB-231	Mef2c- β-catenin	Promotes the invasion of breast cancer cells.	Metastasis	[131]
			miR-124/ -145	Mesenchymal stem cells	Glioma cells and glioma stem cells	SCP-1/Sox2	Decrease the migration of glioma cells and the self-renewal of glioma stem cells.	Proliferation	[132]
			miR-21/ -3a	Bone marrow-derived MSCs	Breast cancer cells	TPM1/PDCD4/ Bcl-2	Elicit pro-tumorigenic and anti-apoptotic effects.	Proliferation	[133]
			miR-200	Metastatic breast cells	Non-metastatic breast cells	ZEB1/ZEB2	Suppress the EMT and enhance the reverse process, mesenchymal-to-epithelial transition (MET) by inhibiting the expression of Zeb1 and Zeb2.	Metastasis	[134]
			MiR-373	breast cancer cells	/	/	downregulate the protein expression of ER and inhibit apoptosis induced by camptothecin.	Malignant prediction	[66]
		Ovarian cancer	miR-200a/b/c/141	SKOV-3 and OVCAR-3	Ovarian cancer cells (OC)	ZEB1 (TCF8/ZFHX1A/δEF1) and ZEB2 (SIP1/ZFHX1B/SMAD1P1)	Down-regulation of miR-200 in mesothelial cells promotes cancer cell attachment and proliferation.	Proliferation	[55]
			let-7 family	SKOV-3	OVCAR-3	/	Exosome release varies between ovarian cancer cell lines and is correlated with invasive potential.	Metastasis	[96]
			miR-21-5p	CP70	A2780	NAV3	Increases platinum-resistance in A2780 cells.	Drug resistance	[135]
			ATF2, MTA1, and ROCK1/2	High-grade ovarian cancer	Endothelial cells	/	Exosomes derived from high-grade ovarian cancer alter angiogenesis compared to non-high-grade ovarian cancer cells.	Metastasis	[136]
			miR-24-3p, −891a, and -106a- 5p	The serum of patients with NPC or TW03 cells	T-cell	MARK1	Alter T-cell proliferation and differentiation.	Metastasis	[137]
			miR-127-3p	/	OVCAR-3 and Caov-3 cells	Bcl-associated athanogene 5 (BAG5) gene	Inhibits the BAG5 gene, and subsequent BAG5 upregulation ameliorated the tumor-suppressive effects of miR-127-3p overexpression in OC.	Proliferation	[138]
				OC ES-2 cells	Endothelial cells	PPP1CA	The upregulation of PPP1CA in OC is attributed to the downregulation of hsa-miR-127-3p.	Proliferation	[139, 140]
			miR-339-5p	OC ES-2 cells	Endothelial cells	WNT (CHD8)	CHD8 inhibits the transcription of β-catenin target genes through chromatin compaction and it may be a tumor suppressor gene. Overexpression of exosomal miR-339-3p could influence WNT3A/CHD8 pathway.	Proliferation and metastasis	[140, 141]
			miR-409-3p	OC ES-2 cells	Endothelial cells	WNT (CTBP1)	CTBP1 was demonstrated to activate the expression of Wnt genes and downregulate their downstream E-cadherin in a TCF-independent manner. Overexpression of exosomal miR-409-3p could influence WNT7A/CTBP1 pathway.	Proliferation and metastasis	[140, 142]
	Male	Prostate cancer (PC)	MiR-141	Bone metastatic PCa cells	Bone cells	NF-κB signaling	Serum exosomal expression of miR-141 were associated with T-classification and metastasis.	Metastasis	[143]
			miR-375	Serum	PC cells	/	miR-375 is associated with recurrent (metastatic) PCa following radical prostatectomy	Metastasis	[111]
			miR-34a	Docetaxel-resistant PC cells	Docetaxel-resistant	B-cell Lymphoma 2	Influences cell response to docetaxel in prostate cancer cells through regulation of anti-apoptotic BCL-2.	Drug resistance	[74]
			miR-125a	DIAPH3-silenced cells	macrophages	AKT1	Suppresses AKT1 expression and proliferation of cancer.	Proliferation	[48]
			miR-290,-378	PC cells	/	/	Overexpression shorten prostate cancer overall survival.	Prognosis	[67]
			miR-1290	Plasma	/	/	Correlation with overall survival	Prognosis	[111]
			miR-19b	Urine	/	/	Correlation with overall survival	Prognosis	
Neural system		Neuroblastoma	miR-21	NBL cells	Human monocytes	TLR8-NF-кB	/	Drug resistance	[143]
			miR-155	Monocytes	NBL cells	TERF1			
Hematological system		Hematological malignancies	miR-210	K562 under hypoxic conditions	Umbilical vein endothelial cells	EFNA3	Exosomal miRNAs derived from cancer cells under hypoxic conditions may affect angiogenic activity in endothelial cells.	Metastasis	[90]
			miR-126	LAMA84	Endothelial cells	CXCL12 and VCAM1	HUVECs with a miR-126 inhibitor reversed the decrease in CXCL12, restores motility and adhesion in LAMA84 cells.	Metastasis	[144]
			miR-202-3p	Chronic lymphocytic leukemia (MEC1)	Human stromal cells	c-Fos and ATM	Enhances proliferation of recipient cells.	Proliferation	[145]
			miR-92a	K562 cells	Umbilical vein endothelial cells	Integrin α5	Enhances endothelial cell migration and tube formation.	Metastasis	[146]
			miR-21	CLL cells	MSCs and endothelial cells	/	Induce differentiation of stromal cells into cancer-associated fibroblasts.	Metastasis	[147]
			miR-135b	Multiple myeloma cells	endothelial cells	FIH-1	Exosomal miR-135b from HR-MM cells enhances endothelial tube formation under hypoxic conditions via the HIF-FIH signaling pathway.	Metastasis, angiogenesis	[148]
Others		Melanoma	miR-125b	PLX4032-resistant melanoma cell line	Primary melanoma cell lines	apoptotic pathways	miRNA inhibitors increased the fraction of apoptotic cells in LM16-R cells	Metastasis	[149]
			miR-31, − 185, and -34b	A375 and SK-MEL-28	Normal melanocytes	HAPLN1, GRP78	/	Metastasis	[150]
			miR-222	Metastatic melanoma cell lines	Primary melanoma cell lines	p27Kip1	Activates the PI3K/AKT pathway.	Metastasis	[151]
		Merkel Cell Carcinoma (MCC)	miR-30a, miR-34, miR-142-3p, miR-1539	MCV-positive or -negative tumors	/	/	Upregulation when discriminating between MCPyV-negative and MCPyV-positive MCCs	MCPyV infection	[63, 152]
			miR-181d				Downregulation when discriminating between MCPyV-negative and MCPyV-positive MCCs		

Proliferation is an important aspect of cancer development and progression that is manifested by altered expression and/or activity of cell cycle-related proteins. Constitutive activation of many signal transduction pathways also stimulates cell growth [46]. miR-584-derived exosomes from HCC cells target TGF-β-activated kinase-1 (TAK1) and associated signaling, leading to TAK1 downregulation. TAK1 is an essential inhibitor of hepatocarcinogenesis and has a direct effect on cancer progression through repression of the telomerase reverse transcriptase gene. That is, miR-584 has an indirect promoting effect on tumor proliferation [47]. Some other findings suggest that miR-125a from TDEs as a result of diaphanous-related formin-3 (DIAPH3) loss or growth factor stimulation may condition the tumor microenvironment through multiple mechanisms, including the proliferation of cancer cells and suppression of tumor-infiltrating immune cells [48]. Still another research showed that miR-1246 packaged in exosomes from 2 Gy-irradiated BEP2D cells could act as a transfer messenger and contribute to DNA damage by directly repressing the DNA ligase 4 (LIG4) gene, which inhibited the proliferation of nonirradiated cells [49].

The stages of tumor proliferation do not have obvious boundaries, and each stage of development does not exist independently. Tumor proliferation is often a contributing factor to the further development of tumor angiogenesis and metastasis.

### Exosomal miRNA and tumor angiogenesis

Tumor angiogenesis comprises several steps: enzymatic degradation of the vessel’s basement membrane, endothelial cells proliferation, migration, sprouting, branching, and tube formation. In tumor microenvironment, exosomes released by different cell types have been shown to function as positive mediators during this process [50], including mesenchymal stem cells, stromal cell, endothelial cells [51].Considerable attention is now focused on the role of miRNAs secreted by TDE acting on the process of vascularization.

Hypoxia is one of the main factors involved in tumor angiogenesis and can affect the activity of various substances and promote expression of exosomal miRNAs. Previous studies have demonstrated that increases in tissue inhibitor of metalloproteinases-1 (TIMP-1) upregulates miR-210 by inducing pro-tumorigenic PI3K/AKT/HIF-1 signaling. Subsequent downregulation of miR-210-targeted proteins results in increased pro-angiogenic properties of exosomes released by TIMP-1-overexpressing cells and thus contributes to a new mode of action by which TIMP-1 can support lung cancer progression [52]. In addition to miR-210, researchers have found that miR-21 in exosomes leads to STAT3 activation, which increases VEGF levels in recipient cells and leads to angiogenesis and malignant transformation of human bronchial epithelial (HBE) cells [53].

### Exosomal miRNA and tumor metastasis

The metastatic process involves manipulation of the cellular microenvironment to optimize conditions for deposition and growth both locally and at a distance [54]. Intercellular communication can occur through various signaling molecules. Many groups have confirmed that tumor-derived exosomes are involved in the different steps of the metastatic cascade. For example, EMT is a complex molecular and cellular process involved in tissue remodeling that has been extensively studied as a facilitator of tumor progression. The miR-200 family inhibits EMT and cancer cell migration by directly targeting the E-cadherin transcriptional repressors ZEB1 and ZEB2 [55].Based on the researches, the mechanism by which miRNAs packaged by TDEs influence tumor metastasis needs to be further explored [56].

Studies have reported four general mechanisms of exosomal miRNA delivery during tumor development in the microenvironment [57]. First, less invasive tumor cells can take up miRNAs delivered from invasive tumor cells via TDEs, which may prompt worsening of a primary tumor. For example, metastatic breast cancer likely promoted cell invasion via release of exosomal miR-10b by the primary tumor into the culture environment of surrounding normal cells. This role of miRNAs packaged by TDEs acting on neighboring cells to transmit a message (produced by a donor cell and taken up by a recipient cell) resembles a paracrine mechanism of intercellular communication [56] (Fig. 2-a).With respect to the second mechanism, primary tumor cells can communicate with other cells via exosomal miRNAs in the tumor microenvironment. For example, by downregulating tight junctions and destroys the barrier function of endothelial monolayers, cancer-secreted miR-105expressed and secreted by metastatic breast cancer cells induces vascular permeability and promotes metastasis [58]. miRNAs have been reported to enter the circulatory system and travel to distant organs to deliver their message by targeting their recipient cells, emphasizing the potential of miRNAs to act as signals involved in preparing a distant site for tumor proliferation [59] (Fig. 2-b). A third mode of communication involves exosomes derived from normal cells or routine biological process that can alter the behavior of tumor cells. For example, after metastasis to the brain, but not to other organs, human and mouse tumor cells regulated by microRNAs from brain astrocytes both lost PTEN expression [50]. Another example is the exosomal level of miR-122-5p was increased upon hepatoma cell damage treated by apoptotic agent and then increased cell mobility by SDC1 downregulation [60].The last mode focus on some tumor caused by viral infections. The cells infected by virus released aberrant quality and quantity of exosomal miRNA, leading more health cells and themselves to precancerous conditions. For example, in the Burkitt Lymphoma Mutu Cell Lines, Epstein-Barr virus (EBV) infection in type III latency modulates the biogenesis of exosomes and expression profile of exosomal miRNAs, such as miR-877 [61], which may contribute to the induction of EBV-associated tumors by modulating cell and virus functions [62].Some other studies showed that Merkel cell polyoma virus seems to be the major causal factor for Merkel cell carcinoma (MCC). By comparing MCPyV positive cells with negative ones, miR-181d as a tumor suppressor was downexpressed in MCPyV-positive cells [63]. (Fig. 2-c).

Organ-specific metastasis is a multi-step and complicated process that includes tumor-host crosstalk among cells as well as communication between cells. Moreover, the crucial role of the tumor environment, including signaling and key molecules required for tumor metastasis, cannot be ignored.

## Exosomal miRNA and clinical implications

### Exosomal miRNA as a predictor of tumor response to treatment

Primary acquired resistance to chemotherapy, radiotherapy and targeted therapies remains a major stumbling block in cancer treatment [64, 65]. The key signaling pathway components in drug response, involving drug targets, transporters, and cell cycle- and apoptosis-related components, include several functional proteins that can be affected by miRNA expression [66]. Exosomes can be regarded as vehicles for loading miRNAs, targeting and combining fundamental genetic molecules in the pathways mediating chemotherapy, radiotherapy and targeted therapies.

Recent studies have reported that treatment of prostate cancer with paclitaxel (PTX) often fails due to the development of chemo-resistance caused by downregulation of the tumor suppressor gene miR-34a. This miRNA has been suggested to be an intracellular and exosomal predictive biomarker for response to docetaxel with clinical relevance to prostate cancer progression by regulating the anti-apoptotic gene BCL-2 [67]. Other researchers have reported that tamoxifen-sensitive breast cancer cells can acquire drug resistance after internalizing exosomes derived from tamoxifen-resistant breast cancer cells. The underlying mechanism involves inhibition of P27 and ERα expression in tamoxifen-sensitive cells by miR-221/222 carried within the transferred exosomes [68]. Furthermore, the research on exosomal miR-21 as biomarker of treatment outcome in non-small cell lung cancer (NSCLC) has also been developed. It was revealed that the high level of miR-21 related to the acquired resistance to the treatment consisting of epidermal growth factor receptor (EGFR) and tyrosine kinase inhibitors (TKIs) [69]. The radio sensitivity mediated by PI3K/Akt pathway represents also an aspect controlled by miR-21, and the inhibition of miR-21 improved the sensitivity to radiotherapy [70], which would be advantages of miR-21 as a useful predictor of the therapeutic response, and constructive, worse outcome [71].

Therefore, some exosomal miRNAs can provide information about the identity of the cell type from which they are derived, the target, and the cellular state, including therapy resistance. Accordingly, it is possible to monitor and regulate tumor resistance, and achieve personalized therapy.

### Exosomal miRNAs as fascinating possibility for tumor biomarker

The cargo of exosomes is specific for the parental cells and the conditions in which they produce them, which implied that circulating miRNAs in exosomes had the potential toserve as prognostic and predictive biomarkers [72]. This review focuses on the biological characteristics of exosomal miRNAs as cancer surrogate biomarkers. Different miRNAs from tumor-related (TR) exosomes have been detected as biomarkers in the plasma of tumor patients.

As the potential role of tumor diagnosis, the results of a meta-analysis suggested that miR-21-containing circulating exosomes, which can also be detected in feces, in plasma may be a reliable and non-invasive biomarker for colorectal cancer diagnosis [73]. Moreover, recent studies have claimed that circulating exosomal miRNA-373 is upregulated in receptor-negative breast cancer patients [74]. Additionally, miR-1290 and miR-375 upregulation might indicate poor overall survival in castration-resistant prostate cancer [75], and exosomal miR-19a cluster expression level in serumarecorrelated with recurrence in colorectal cancer [76].

In addition to tumor markers, exosomal miRNAs can also act as tumor development inhibitors, with a fascinating possibility for tumor therapy. The correlation between miRNAs from TDEs and immunology is ubiquitous, further demonstrating differences between tumor phenotypes. Thus, secreted miRNAs may be considered a type of immune cell effector. For example, transfer of miR-142 and miR-223 influences post-transcriptional regulation of proteins in HCC, including decreased expression of reporter proteins and endogenously expressed stathmin-1 and insulin-like growth factor-1 receptor. This ultimately inhibits proliferation of these cancerous cells, suggesting that miR-142 and miR-223 may act as inhibitors of tumor treatment [77]. Furthermore, exosome-derived miR-29c induces apoptosis in bladder cancer cells by downregulating BCL-2 and MCL-1 [78], and some exosomal miRNAs, such as miR-127 and miR-197, can elicit dormancy in tumor metastasis and proliferation, decreasing proliferation and eliciting dormancy in bone marrow metastasis of breast cancer. All of these molecules may inhibit tumor treatment [79]. To utilize exosomal miRNAs as a diagnosis biomarker, a fluorescent system with antidisturbance should be developed, due to the complex biosystem. Surprisingly, because of the self-referencing capability, ratiometric fluorescent measurement is able to cancel out environmental fluctuations by calculating the emission intensity ratio at two different wavelengths [80]. Recently, the practical applications of ratiometric fluorescent bioprobes has been improved. For instance, a ratiometric fluorescent bioprobe based on DNA-labeled carbon dots (DNA-CDs) and 5,7-dinitro-2-sulfo-acridone (DSA) coupling with the target-catalyzing signal amplification for the detection of exosomal miRNA-21. There was high fluorescence resonance energy transfer (FRET) efficiency between carbon dots (CDs) and DSA when the bioprobe was assembled [81].

To date,there is increasing evidence for the roles of TDEs. Considering that compared with total circulating RNAs, exosomes typically target specific cells, detection of exosomal miRNAs in clinical examination appears reasonable, which might assist physicians with predicting cancer prognosis [82].

### Exosomal miRNA delivery system: Opportunities and challenges

In previous researches, miRNAs encased in TDEs are more likely to escape attack by immune systemand able to cross the blood-brain barrier. Moreover, exosomes are likely to protect their cargo from clearance or damage by the complement fixation or macrophages due to their double-layered membrane and nanoscale size, thus prolong their circulation half-life and enhancing their biological activity [83]. Exosomes can deliver miRNAs to target recipient cells with a distinctcomposition of proteins and lipids on their surface. In addition, exosome membrane is rich in sphingomyelin, ceramide, and cholesterol, which help to distinguish exosomes from the cell membrane and facilitates their uptake by recipient cells. As a consequence, exosomes always succeed, even though they sometimestake a longer path to reach their target [84].

Differential experimental skills have been employed in an attempt to purify reticulocyte exosomes from tissue culture medium, and new methods for exosome purification were developed to reduce the cell media required,thus enhancing maneuverability and improving efficiency [85]. In addition, the abovementioned studies have found that Dicer and Ago2, the key components of miRNA processing, are functionally present in exosomes, suggesting that miRNA might not be the only cargo carried by EVs [18]. Another challenge is how to load the desired cargo. For example, miRNA can be efficiently encapsulated into exosomes by manipulating exosome-producing cells to overexpress cargo miRNA. By usinga cell-specific protein present in the membrane of the exosomes,these encapsulated miRNAs were delivered to EGFR-expressing breast cancer cells. However, researchers were unable to encapsulate miRNA into HEK-293-derived exosomes using electroporation [86]. In addition to technological issues, exosomes have the potential to spread numerous pathogens. Many pathogenic factors, including viral proteins and fragments of viral genomes, can be incorporated into exosomes derived from virus-infected cells, and exosome-mediated delivery of these factors has been shown to affect the immune responses to infection and to modulate recipient cells responses. For example, HIV-1 achieves cell entry via exosome-mediated transfer of chemokine receptor 5 to recipient cells [87]. There are still some limitationsregarding encapsulated miRNAs in the exosomal miRNA delivery system. As cell-based delivery vehicles, exosomes sufficiently deliver their functional message to recipient cells without negative side effects; thus, exosomes are attracting attention in molecular medicine as potential modulators of disease-mediated processes. Nevertheless, we cannot ignore the problems of miRNA in itself, some have shown that imported miRNA results in little cellular toxicity and has substantial effects on miRNA regulation in recipient cells, for example, exosomal transfer of miR-155 inhibitors and mimics to macrophages [88].

Currently, a growing number of evidence reveals that exosomal miRNAs were highly disease-related not only with tumor but other diseases, and both sides will improve prominently by interoperability of knowledge. For example, miR-21-5p, miR-29a-3p and miR-126-3p are involved in pathways related to diabetic kidney disease (DKD) pathogenesis, such as apoptosis, fibrosis, and extracellular matrix accumulation. They seem to be dysregulated in patients with different stages of DKD, constituting potential biomarkers of this disease [89]. At the same time, miR-21 and miR-29a act in NSCLC tumor growth and metastasis [41], and miR-126 promotes the Hematological malignancies metastasis [90]. Some other studys showed that high-glucose(HG) exosomes contained high levels of miR-28, miR-31a, and miR-130acompared to exosomes derived from non-HG-stimulated Schwann cells, which might promote development of diabetic peripheral neuropathy. Schwann cells are the most abundant myelinated cells [91]. Herein, a potential point on whether the high release of these exosomal miRNA would influence tumor development need to be explored. Some other study held that the transport of miRNAs, within or in association with exosomes, may provide a distant and potentially more bioactive pool of circulating miRNAs compared to those that are riboprotein bound. Currently, however, there is no evidence to suggest functional differences between exosomal miRNAs and free ones, nor is it known whether exosomal and free miRNAs are differentially regulated in response to stimulation. Future studies need to classify whether miRNA packaging into exosomes and exosomal uptake is a selective/stimulus dependent process [92].

Thus, applying exosomal miRNAs to clinical treatment is a challenging but intriguing endeavor that requires further exploration by researchers and clinicians.

## Conclusion

To successfully develop advanced therapeutic options for the treatment of cancer, exosomal miRNAs should not be disregarded. Based on the specific function of miRNA delivered by TDE, we will be able to counteract pro-tumorigenic and pro-metastatic signals that contribute to the growth, spread, and drug resistance of tumor cells by potentially engineering the miRNA and protein cargo of exosomes or by interfering with their trafficking.However, further study is required to cause tumor cells to forsake “heresy” and return to the “truth”.Therefore, future efforts should focus on identifying the right correct of TDE-mediated immune escape and TDE-mediated tumor resistance to avoid the disadvantages of exosomal miRNAs [93]. Moreover, an effective selective mechanism for exosomal miRNA delivery system and technologies for miRNA mimic-importing TDEs can also be expected.

## Abbreviations

3′UTR: 3′ untranslated region
AGO2: Argonaut
CAFs: Cancer-associated fibroblasts
CDs: Carbon dots
CRC: Colorectal cancer
CXC: Chemokine
CXCR4: Chemokinereceptor 4
DCs: Dendritic cells
DIAPH3: Diaphanous-related formin-3
DKD: Diabetic kidney disease
DNA-CDs: DNA-labeled carbon dots
DSA: 5,7-dinitro-2-sulfo-acridone
EBV: Epstein–Barr virus
ECM: Extracellular matrix
ED: Editing
EGFR: Epidermal growth factor receptor
EMT: Epithelial-to-mesenchymal transition
ESCC: Esophageal cancer
ESCRT Complex: Endosomal sorting complex required for transport
FRET: Fluorescence resonance energy transfer
HBE: Bronchial epithelial
HCC: Hepatocellular carcinoma
LSPR: Localized surface plasmon resonance
MCC: Merkel cell carcinoma
MCC: Merkel cell carcinoma
MET: Mesenchymal-to-epithelial transition
miRNAs: MicroRNAs
mRNA: Messenger RNA
MVB: Multivesicular bodies
NFs: Fibroblasts
NK: Natural killer cells
NSCLC: Non-small cell lung cancer
nSMase2: Sphyngomyelinase 2
PC: Pancreatic cancer
pri-miRNAs: Primary miRNAs
PTX: Paclitaxel
qRT-PCR: Quantitative reverse transcription polymerase chain reaction
RFXAP: Regulatory factor X-associated protein
RISC: RNA induced silencing complex
RPM: Reads per million reads mapped to miRNAs
SERS: Surface-enhanced Raman scattering
TAK1: TGF-βactivated kinase-1
TDEs: Tumor-derived exosomes
TKIs: Tyrosine kinase inhibitors
TLRs: Toll-like receptors
VEGF: Vascular endothelial growth factor
WT: Wild-type
